# Novel macrophage-related gene prognostic index for glioblastoma associated with M2 macrophages and T cell dysfunction

**DOI:** 10.3389/fimmu.2022.941556

**Published:** 2022-09-13

**Authors:** Hang Ji, Zhihui Liu, Fang Wang, Haogeng Sun, Nan Wang, Yi Liu, Shaoshan Hu, Chao You

**Affiliations:** ^1^ Department of Neurosurgery, West China Hospital, Sichuan University, Chengdu, China; ^2^ Cancer Center, Department of Neurosurgery, Zhejiang Provincial People’s Hospital, Hangzhou Medical College, Hangzhou, China

**Keywords:** glioblastoma, tumor microenvironment, tumor-associated macrophage, immune checkpoint inhibition, eicosanoid metabolism

## Abstract

This study aims to construct a Macrophage-Related Gene Prognostic Index (MRGPI) for glioblastoma (GBM) and explore the underlying molecular, metabolic, and immunological features. Based on the GBM dataset from The Cancer Genome Atlas (n = 156), 13 macrophage-related hub genes were identified by weighted gene co-expression network (WGCNA) analysis. 5 prognostic genes screened by Kaplan-Meire (K-M) analysis and Cox regression model were used to construct the MRGPI, including GPR84, NCF2, HK3, LILRB2, and CCL18. Multivariate Cox regression analysis found that the MRGPI was an independent risk factor (HR = 2.81, CI95: 1.13-6.98, p = 0.026), leading to an unfavorable outcome for the MRGPI-high group, which was further validated by 4 validation GBM cohorts (n = 728). Thereafter, the molecular, metabolic, and immune features and the clinical implications of the MRGPI-based groups were comprehensively characterized. Gene set enrichment analysis (GSEA) found that immune-related pathways, including inflammatory and adaptive immune response, and activated eicosanoid metabolic pathways were enriched in the MRGPI-high group. Besides, genes constituting the MRGPI was primarily expressed by monocytes and macrophages at single-cell scope and was associated with the alternative activation of macrophages. Moreover, correlation analysis and receiver operating characteristic (ROC) curves revealed the relevance between the MRGPI with the expression of immune checkpoints and T cell dysfunction. Thus, the responsiveness of samples in the MRGPI-high group to immune checkpoint inhibitors (ICI) was detected by algorithms, including Tumor Immune Dysfunction and Exclusion (TIDE) and Submap. In contrast, the MRGPI-low group had favorable outcome, was less immune active and insensitive to ICI. Together, we have developed a promising biomarker to classify the prognosis, metabolic and immune features for GBM, and provide references for facilitating the personalized application of ICI in GBM.

## Introduction

Glioblastoma (GBM) is the most common and devastating primary brain tumor that possess a desperate outcome, with median survival remaining around 15 months after standard treatment (1–3). Developing robust biomaerkers for prognosis and therapies remains challenging. Novel tumor therapies including immune checkpoint inhibition (ICI) aim to block immune checkpoint signaling pathways, such as the PD-1/PD-L1 axis and CTLA4, which bring remarkable survival benefits for several malignancies (4–7). Yet, the application of ICI in GBM is limited, which may partially ascribe to the immune-suppressive tumor microenvironment (TME) (8, 9). Tumor-associated macrophages (TAMs) in GBM refer to blood-derived monocytes/macrophages and intrinsic microglia, and the alternatively activated TAMs orchestrate an immunosuppressive TME thus impeding the anti-tumor immune activity (10–12). Interstingly, increasing evidence suggests that TAMs involve in the expression of immune checkpoints in the TME and play a vital role in inducing CD8 T lymphocyte dysfunction (11, 13–15). Given that GBM is a class of TAMs-rich tumors (16), the identification of biomarkers associated with alternative activation of TAMs may provide prognostic convenience for GBM on the one hand, and pave the way for the application of ICI on the other.

Although TAMs can be simply divided into M1 and M2 phenotypes, the molecular features characterizing the functional status of TAMs in the GBM TME remain loosely defined. Unlike the usual dogma, MARCO may be a transcriptomic marker for TAMs in GBM (17). In addition, STAT3 signaling is responsible for the polarization and immunosuppressive functions of macrophages and microglia in the GBM microenvironment instead of STAT6 (11, 18). Therefore, the identification of biomarkers associated with alternative activation of macrophages needs to take into account the specific immune context of the central nervous system. Recently, great advances have been made in the study of the spectrum of the functional state of macrophages based on the transcriptome (19, 20), which resolved the molecular functional networks associated with the different polarization states of alveolar macrophages. Building on these achievements, we have the opportunity to identify the gene signatures associated with the alternative activation of TAMs in GBM and to explore their clinical significance.

From this perspective, we developed a macrophage-related gene prognostic index and explore its molecular underpinnings and clinical implications. We started with the differentially expressed macrophage-related genes in the GBM expression profile and screened out the hub genes of prognostic significance and constructed the MRGPI. Then, we comprehensively explored the molecular, metabolic, and immunological features of MRGPI at bulk tumor, single-cell transcriptome, and protein levels and identified the expression patterns of immune checkpoints and the functional states of TAMs associated with MRGPI. Based on several machine learning algorithms, the association between the MRGPI with ICI responsiveness of GBM samples were also revealed. Overall, our study not only provide a biomarker of clinical implications, but also offer some insights into understanding the cancer biology of GBM.

## Materials and methods

### Data collection and pre-processing

RNA-seq data of 173 GBM samples and corresponding demographics were retrieved from The Cancer Genome Atlas (TCGA) database (https://portal.gdc.cancer.gov). 156 GBM samples remaining after exclusion of normal and formalin-fixed samples and was difined as the training data set. 4 additional GBM cohorts were collected and used as the validation data sets, including CGGA325 (n = 139), CGGA693 (n = 249), Rembrandt (n = 181), and Gravendeel (n = 159). RNA-seq data of integrated GBM and LGG dataset (n = 702) and corresponding demographics were downloaded from the UCSC Xena data portal (https://xenabrowser.net/). RNA-seq data of 214 normal brain tissue samples (cortex) were retrieved from the UCSC Xena data portal (http://xena.ucsc.edu/). The batch effect was eliminated using the R package ‘sva’. Count value was converted to TPM for regression analysis, GSEA, and comparison of gene expression at the bulk-tumor level. The macrophage bona fide gene (BFG) list was summarized by Xue J. et al (19). Single-cell RNA-seq datasets including GSE131928 and GSE70630 were retrieved from the TISCH data portal (http://tisch.comp-genomics.org/) (21). The expression profile of 30 types of TCGA cancers was integrated by Thorsson et al. (16), and corresponding demographics were retrieved from the UCSC Xena data portal. The transcriptome subtype of TCGA GBM samples was summarized by Wang et al. (22).

### Identification of macrophage-related hub genes and constriction of MRGPI

Based on the merged TCGA GBM and GTEx normal tissue RNA-seq expression profile, the differentially expressed genes (DEGs) were estimated using the R packages ‘limma’ and ‘edgeR’ (|log_2_FC| >= 0.5, adj p-val < 0.05) (23, 24). After intersecting the macrophage BFGs with the DEGs, differentially expressed macrophage-related genes were obtained and annotated using functional enrichment analysis based on the webtool Metascape (http://www.metascape.org/) (25). WGCNA analysis was performed to identify hub genes (26). Briefly, the similarity matrix and adjacency matrix (signed) were constructed sequentially based on the expression profile of differentially expressed macrophage-related genes, and the soft threshold of β was calculated. Then, the adjacency matrix was transformed into the topological matrix and the dynamic pruning tree was built to identify the gene modules with a merging threshold function at 0.25. Genes involved in the module brown and turquoise were candidates for K-M analysis to determine prognostic significance. Further, univariate and multivariate Cox regression analysis was performed to determine the independent prognostic significance of these genes. To reveal the regulatory mechanism of genes of independent prognostic significance, related transcriptional factors (TFs) and miRNA interaction network was constructed using the webtool NetworkAnalyst (https://www.networkanalyst.ca/) (27). The regression coefficients of genes of independent prognostic significance were determined using multivariate Cox regression analysis. The MRGPI was defined as the sum of gene expression multiplied by its multivariate regression coefficient. Samples were then split into MRGPI-high and MRGPI-low groups by the median value. The web tool TISCH was employed to identify the cellular location of selected genes.

### Immunohistochemistry for genes comprising MRGPI

All samples were obtained according to the protocol approved by the Ethics Review Committee of Zhejiang Provincial People’s Hospital. All subjects were given written informed consent to participate. Formalin-fixed, paraffin-embedded tumor tissues (approximately 0.5cm×0.5cm×0.2cm) were collected during surgical excision and were further divided into core and margins by two senior neurosurgeons. The procedure for immunohistochemical staining has been described before (28). Briefly, after heat-induced antigen retrieval, tumor sections were stained with a 1:500 dilution of the corresponding antibody against CCL18 (22303-1-AP, Proteintech, Wuhan Sanying, China), GPR84 (DF2769, Affinity Biosciences, China), HK3 (13333-1-AP, Proteintech, Wuhan Sanying, China), LILRB2 (DF9604, Affinity Biosciences, China), and NCF2 (15551-1-AP, Proteintech, Wuhan Sanying, China). The staining intensity of the tissue sections was calculated by the IHC profiler plugin of the imageJ software. We selected 4-5 tissue sections from the 3 pairs of tumors and peritumoural tissues for immunohistochemical staining and calculated IHC scores. The score of each section was assigned as the sum of 4 multiplied by the proportion of the strong positive pixels, 3 multiplied by the proportion of the positive pixels, 2 multiplied by the low positive pixels and 1 multiplied by the negative pixels, as described in the original study (29).

### Exploring the molecular and immune characteristics and ICB responsiveness of MRGPI

Gene set enrichment analysis (GSEA) software (v4.2.3) was employed to assess enriched signaling pathways based on the Molecular Signature Database (http://www.gsea-msigdb.org/gsea/msigdb/v7.5.1) (FDR-q < 0.1) (30, 31). Then, genes involved in the WP eicosanoid synthesis pathway were candidates for the multivariate regression model to determine their association with MRGPI. The fraction of 22 immune infiltrations was estimated using the CIBERSORT algorithm (32). Samples with p-value < 0.05 and cells with 0 value in over half of the samples were filtered. To address the association between the functional state of macrophages and MRGPI, single-cell RNA-seq datasets GSE131928 and GSE70630 were included and the R package ‘Seurat’ was employed to dissect these datasets (33–35). Briefly, cells with abnormal gene numbers and ribosome ratios were filtered, and the effect of cell cycle-related genes on clustering was excluded using regression analysis after the identification of highly variable genes. Clustering was performed at a resolution of 0.5. The non-malignant cells were determined using marker genes summarized by the CellMarker database (http://bio-bigdata.hrbmu.edu.cn/CellMarker/) and Neftel et al. (34, 36). For instance, macrophages marker genes include CD14, AIF1, FCER1G, FCGR3A, TYROBP, CSF1R, T cell marker genes include CD2, CD3D, CD3E, CD3G, and marker genes of other cells (mainly oligodendrocytes) include MBP, TF, PLP1, MAG, MOG, CLDN11. The online tool TIDE algorithm was performed to predict sample responsiveness to ICI (http://tide.dfci.harvard.edu/) (37). The TIS score was calculated as an average of log-scale normalized expression of the 18 signature genes associated with interferon-gamma. Besides, the submap algorithm was employed to correct the predicted results with a default parameter (https://cloud.genepattern.org/gp/pages/index.jsf) (38). To evaluate the association between MRGPI and genes related to T cell function, 4 published studies of tumor immune evasion were employed and the hub genes have been collected and screened by Peng et al. Briefly, we calculated the Spearman rho for each positive and negative hit gene and excluded those with insignificant rho with the MRGPI. When measuring the performance of the MRGPI in predicting the positive and negative hits using ROC, positive hits were marked as 1 and negative hits 0.

### Statistics

All statistics were performed using the R software (v4.1.2). Wilcox test was performed to compare continuous variables between groups (t-test for normally distributed variables). K-M survival analysis with the log-rank test was performed to classify survival differences. Univariate and multivariate Cox regression analysis was conducted to determine the independent prognostic value of the variables. The composition ratios were compared using the Chi-square test or Fischer’s exact test. ROC and corresponding AUC were employed to evaluate the association between MRGPI and genes related to T cell function. A two-sided p-value < 0.05 was considered significant. In the GSEA analysis, FDR-q < 0.1 was considered significant.

## Results

### Workflow of the research

**Figure 1:**
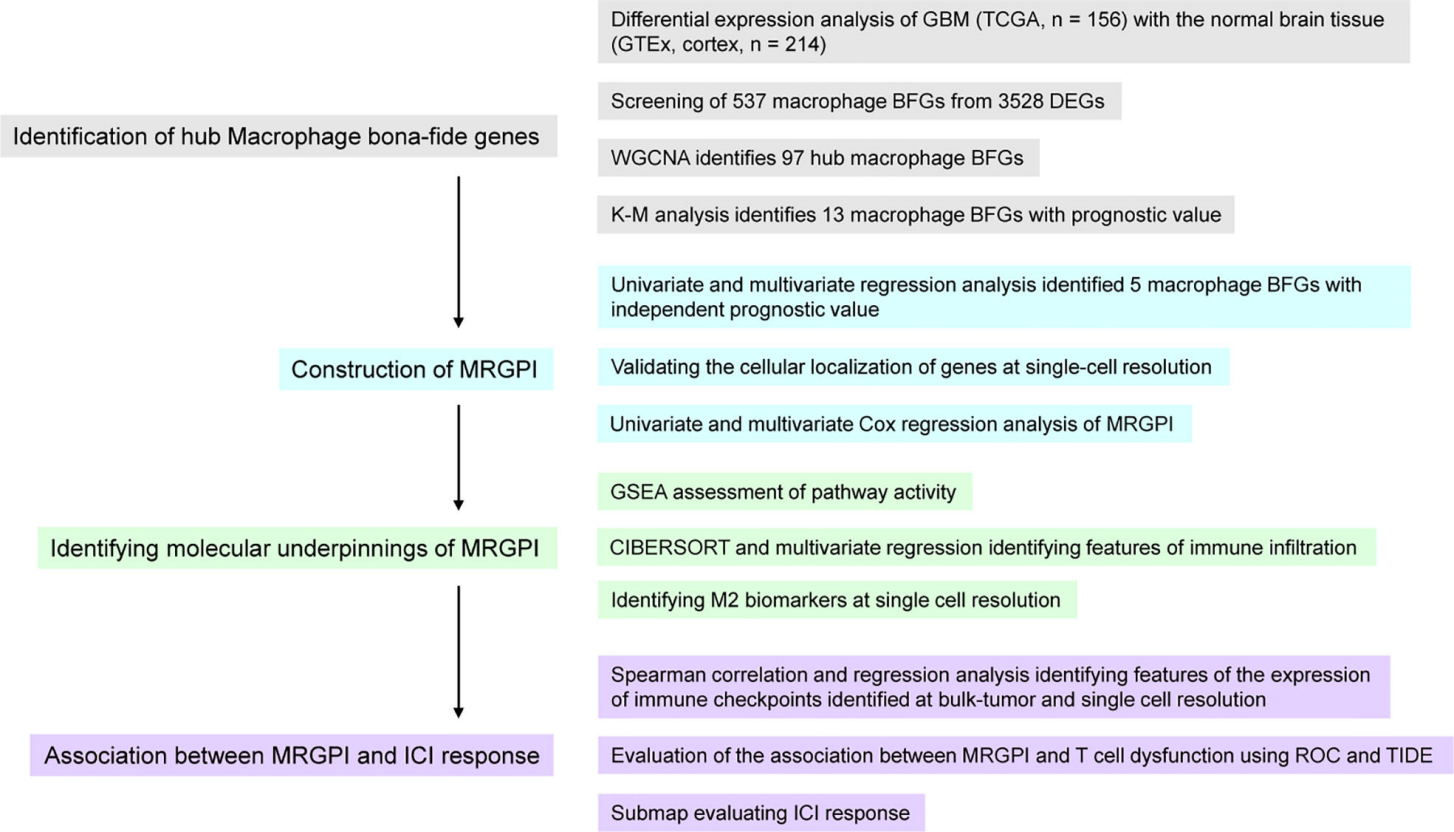
Overview of the workflow of this study.

This study was divided into 4 parts (**Figure 1**). We first identified genes that were aberrantly expressed in GBM by differential analysis and then intersected these DEGs with macrophage BFGs to obtain macrophage-associated genes. Macrophage-associated hub genes were further screened by WGCNA and those genes significantly associated with GBM prognosis were identified by K-M analysis as candidates for subsequent Cox analysis. Next, genes that were statistically significant in the univariate Cox analysis were injected into the univariate Cox analysis, and the resulting five genes and their Cox regression coefficients constituted the MRGPI. We then explored the immune features associated with MRGPI. CIBERSORT and GSEA were conducted to identify characteristics of immune infiltration and immune-related signalling pathways and their relevance with MRGPI. Finally, we employed ROC analysis, the TIDE algorithm, a previously well-constructed TIS score, and the Submap algorithm to assess the association between MRGPI and T cell dysfunction as well as the potential of MRGPI to predict ICI responses.

### Identification of Macrophage-related hub genes

Differential expression analysis was performed to identify macrophage-related hub genes. A total of 3528 DEGs were identified between the GBM sample (n = 156) and normal controls from the GTEx (n = 214), including 1775 upregulated DEGs and 1753 downregulated DEGs (**Supplementary Figure 1A**). Intersecting these DEGs with 9498 macrophage BFGs resulted in 537 differentially expressed macrophage-related genes, of which 416 genes were upregulated and 121 were downregulated (**Supplementary Figure 1B**). Functional enrichment analysis of the 537 genes found that these genes were preferentially enriched in signaling pathways associated with inflammation, cytokine production as well as leukocyte activation (**Supplementary Figure 1C**).

To identify the macrophage-related hub genes, WGCNA analysis was performed on the 537 candidate genes. Setting the correlation coefficient between the log(k) of a node with connectivity log(P(k)) of that node over 0.9, the estimated optimal soft threshold power for a scale-free network was 16 (**Supplementary Figure 2A**). Thereafter, 4 modules were identified based on the optimal soft-thresholding power (**Supplementary Figure 2B, C**). Based on the Pearson correlation coefficient between the module and clinical features, module brown and turquoise were significantly correlated with GBM (cor = 0.8 and 0.69, respectively). A total of 232 genes were included in module brown (n = 54) and turquoise (n = 178). Functional enrichment analysis showed that genes in module brown were mainly enriched in pathways associated with cell proliferation and division, and genes in module turquoise were enriched in pathways related to cell activation and inflammation (**Supplementary Figure 3**). Setting the adjacency threshold for including edges to 0.2, 97 out of 232 genes were defined as hub genes, and their module membership (MM) values and connectivity (within (Kwithin) and outside (Kout) of a module) were summarized in **Supplementary Table 1**. K-M analysis found that 13 of them were of prognostic significance in GBM patients (**Supplementary Figure 4**). Besides, the molecular characteristics of the 13 differentially expressed macrophage-related hub genes were explored. In regulatory network, there were 83 interacting pairs between the hub genes and TFs (**Supplementary Figure 5A**) and 94 interacting pairs between the hub genes and miRNAs (**Supplementary Figure 5B**). To validate the cellular expression of these genes at single cell resolution, multiple single-cell expression profiles were integrated. As a result, the 5 genes were significantly upregulated in the Mono/Macro cluster, both in glioma and a pan-cancer scale (**Supplementary Figure 6A, B**).

### Prognostic significance of the MRGPI

**Figure 2:**
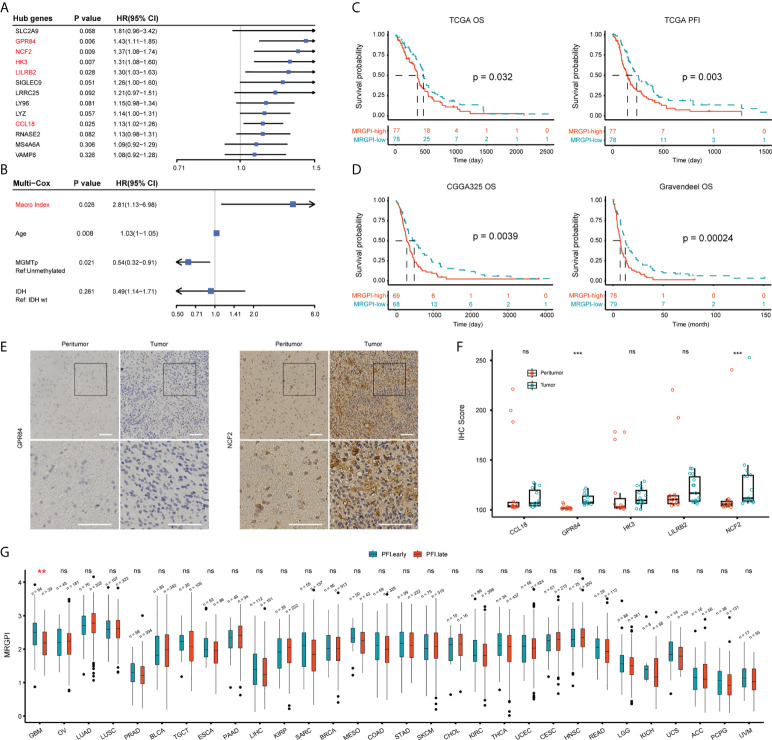
Prognostic significance of MRGPI-based groups. **(A)** Multivariate Cox regression analysis of 13 differentially expressed macrophage-related hub genes. **(B)** Cox regression analysis of the MRGPI and clinicopathological parameters based on the TCGA cohort. Covariates including age, gender, IDH mutation, ATRX mutation, MGMT promoter methylation status, TERT promoter mutation, and MRGPI were included in the initial univariate Cox regression. Covariates with p-values less than 0.01 were further included in the multivariate Cox model. **(C, D)** K-M analysis of the survival and tumor progression-free interval differences between MRGPI-based groups based on TCGA, CGGA325, and Gravendeel GBM cohorts. **(E, F)** Immunohistochemical staining of five genes at the protein level. The tissue was divided into core of the tumor (Tumor) and the margin containing infiltrating tumor cells (Peritumor) in three patients with a pathological diagnosis of GBM. The intensity of staining for the proteins encoded by these genes at the tissue level ranged from negative to positive, with E showing genes with significantly higher IHC scores in the tumor core. The IHC scores of the five genes were shown in F (Scale bar, 100μm). **(G)** Pan-cancer-based MRGPI prognostic significance. Samples were split into early (PFI < 6 months) and late (PFI > 12 months) relapse groups based on PFI. OV, Ovarian serous cystadenocarcinoma; LUAD, Lung adenocarcinoma; LUSC, Lung squamous cell carcinoma; PRAD, Prostate adenocarcinoma; BLCA, Bladder urothelial carcinoma; TGCT, Testicular germ cell tumors; ESCA, Esophageal carcinoma; PAAD, Pancreatic adenocarcinoma; LIHC, Liver hepatocellular carcinoma; KIRP, Kidney renal papillary cell carcinoma; SARC, Sarcoma; BRCA, Breast invasive carcinoma; MESO, Mesothelioma; COAD, Colon adenocarcinoma; STAD, Stomach adenocarcinoma; SKCM, Skin cutaneous melanoma; CHOL, Cholangiocarcinoma; KIRC, Kidney renal clear cell carcinoma; THCA, Thyroid carcinoma; UCEC, Uterine corpus endometrial carcinoma; CESC, Cervical squamous cell carcinoma and endocervical adenocarcinoma; HNSC, Head and neck squamous cell carcinoma; READ, Rectum adenocarcinoma; LGG, Lower grade glioma; KICH, Kidney chromophobe; UCS, Uterine carcinosarcoma; ACC, Adrenocortical carcinoma; PCPG, Pheochromocytoma and paraganglioma; UVM, Uveal melanoma. ***p < 0.001. ns, non significant.

Next, we determined the independent prognostic significance of the 13 differentially expressed macrophage-related hub genes using the univariate and multivariate Cox regression analysis. As a result, GPR84, NCF2, HK3, LILRB2, and CCL18 significantly affected the overall survival (OS) of GBM patients (multivatiate Cox p < 0.05) (**Figure 2A**), of which the protein level of GPR84 and NCF2 was significantly increased in the tumor core region (**Figure 2E, F**, **Supplementary Figure 6C**). Thereafter, a prognostic index (MRGPI) for GBM samples was constructed based on the regression coefficients derived from multivariate Cox analysis (0.2598, 0.1038, 0.0885, -0.1356, 0.0652 for GPR84, NCF2, HK3, LILRB2, and CCL18), and samples were split into MRGPI-high and -low groups by the median value. The clinical features associated with the MRGPI-based groups were summarized in **Table 1**. Notably, multivariate Cox regression analysis identified that MRGPI was an independent prognostic risk factor (HR = 2.81, CI95: 1.13-6.98, p = 0.026) in the TCGA cohort after adjusted for covariates, including age, sex, IDH mutation, MGMT promoter methylation, ATRX mutation and TERT promoter mutation (**Figure 2B**), as well as in validation data sets (**Table 2**). Nevertheless, ROC curves and corresponding area under curves (AUCs) suggested that the MRGPI provided a mediocre prediction performance (**Supplementary Figure 7A**). Thus, the MRGPI-high group had decresed OS and progression-free interval (PFI) in the TCGA, and in the CGGA325 and Gravendeel cohorts (**Figure 2C, D**). Moreover, 30 types of cancers in the TCGA project were included and samples were divided into early relapse (PFI < 6 months) and late relapse (PFI > 12 months) groups. Only in the GBM sample was there a significant difference in MRGPI between the two groups (**Figure 2G**).

**Table 1:** Clinical features associated with MRGPI.

	MRGPI-high	MRGPI-low	p-value
**Age**			
>= 60	40 (54.79%)	38 (49.35%)	
< 60	33 (45.21%)	39 (50.65%)	0.518
**Gender**			
Male	47 (64.38%)	53 (68.83%)	
Female	26 (35.62%)	24 (31.17%)	0.606
**IDH status**			
Mutation	3 (4.2%)	8 (10.96%)	
Wildtype	69 (95.83%)	65 (89.04%)	0.208
**MGMT promoter**			
Methylated	17 (32.08%)	30 (46.88%)	
Unmethylated	36 (67.92%)	34 (53.13%)	0.131
**Transcriptome subtype**			
ME	51 (71.83%)	12 (19.05%)	
PN	3 (4.23%)	15 (23.81%)	
CL	13 (18.31%)	33 (52.38%)	
NE	4 (5.63%)	3 (4.76%)	8.876E-10

ME, mesenchymal; PN, proneural; CL, classical; NE, neural.

**Table 2:** Cox regression analysis of the MRGPI in validation data sets.

Cohort	Type	Sample size	Covariates	Uni-Cox			Multi-Cox			
				HR	CI95	P value	HR	CI95	P value	
TCGA	RNA-seq	n = 156	Age, Gender, IDH mutation, ATRX mutation, TERT promoter mutation, MGMT promoter methylation	2.75	1.40-5.38	0.003	2.81	1.13-6.98	0.026	
CGGA325	RNA-seq	n = 139	Age, Gender, IDH mutation, MGMT promoter methylation, Primary/Recurrent,1p19q co-deletion	1.87	1.26-2.78	0.002	2.07	1.36-3.17	0.001	
CGGA693	RNA-seq	n = 249	Age, Gender, IDH mutation, MGMT promoter methylation, Primary/Recurrent,1p19q co-deletion	1.20	0.87-1.66	0.275	NA	NA	NA	
Gravendeel	Microarray	n = 159	Age, Gender, EGFR amplification, IDH1 mutant	2.48	1.55-3.98	2e-4	2.16	1.05-4.41	0.036	
Rembrandt	Microarray	n = 181	Age, Gender, 1p19q co-deletion	1.04	0.43-2.52	0.93	NA	NA	NA

We also explored the prognostic value of MRGPI for LGG. Samples in the TCGA (n = 525), CGGA325 (n = 172), CGGA693 (n = 420), Rembrandt (n = 123), and Gravendeel (n = 116) were included in our study. Consistently, the MRGPI-high group had a significantly decreased OS and PFI (**Figure S7B-G**), indicating that the MRGPI-high robustly predicted an unfavorable outcome of LGG.

### Molecular underpinnings associated with MRGPI-based group

**Figure 3:**
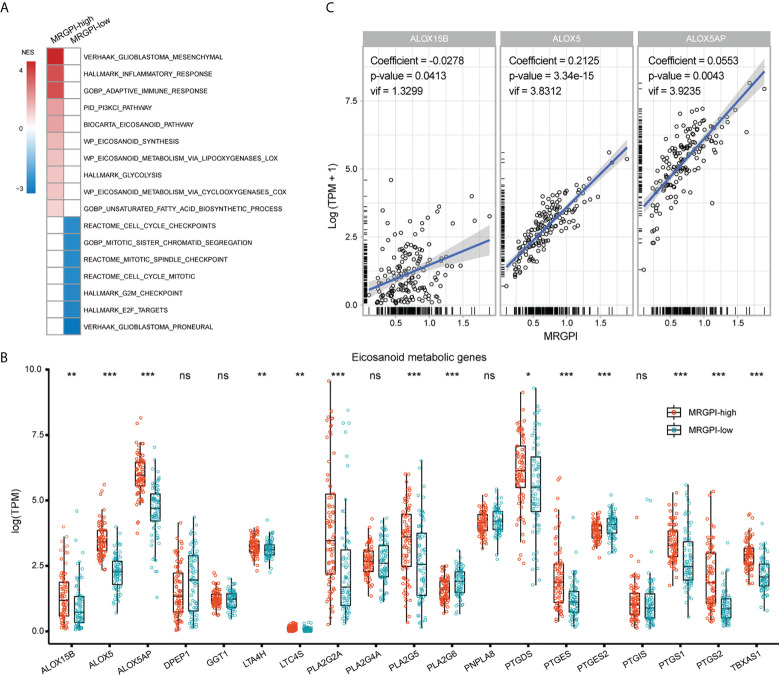
Molecular underpinnings associated with the MRGPI-based groups. **(A)** GSEA analysis of signaling pathways enriched in each group. Pathways of interest with FDR-q < 0.1 were exhibited. The color of the box was proportional to the NES. **(B)** The expression pattern of genes involved in the eicosanoid metabolic pathway between groups. **(C)** Multivariate Cox regression analysis of genes involved in the eicosanoid pathway for the estimation of their correlation with the MRGPI. *p < 0.05, **p < 0.01, ***p < 0.001. ns, non significant.

Then, the molecular underpinnings underlying the MRGPI were explored. GSEA analysis revealed an enrichment of the ME subtype in the MRGPI-high group (**Figure 3A**; **Supplementary Table 2**), which accounted for approximately 72% of the MRGPI-high group. Besides, the MRGPI-high group had activated immune response and altered fatty acid and glucose metabolism (FDR-q < 0.1). On the other hand, the MRGPI-low group had enriched PN subtype gene signature and significant alterations in cell cycle-related signaling pathways. Dysregulated fatty acid metabolism is associated with the biosynthesis of eicosanoids and derivatives and has profound impacts on the immunological features of the TME (39, 40). In terms of the expression pattern of genes involved in eicosanoid metabolism, ALOX5, ALOX5AP, ALOX15B, PTGS1/2, and TBXAS1 were significantly upregulated in the MRGPI-high group (**Figure 3B**), implying an association between the immune response and the activated eicosanoid metabolic pathways in the MRGPI-high group. Furthermore, in the multivariate regression analysis, the expression of ALOX5 and ALOX5AP was proportional to the MRGPI, whereas the opposite was true for ALOX15B (**Figure 3C**). Therefore, these results highlighted that the LOX pathway, especially ALOX and ALOX5AP may play a role in the TME of the MRGPI-high group.

### Immune characteristics associated with MRGPI

**Figure 4:**
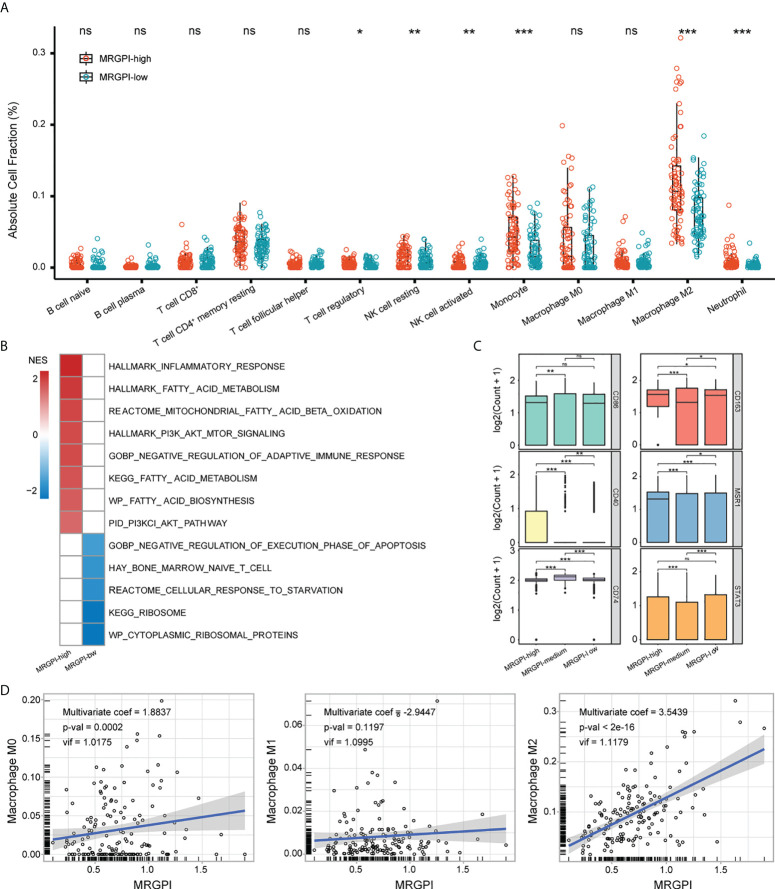
Immune characteristics of MRGPI groups. **(A)** The fraction of immune infiltration was estimated by CIBERSORT. **(B)** GSEA analysis of pathways enriched in each group based on the expression profile of the Mono/Macro subcluster. Pathways with FDR-q < 0.1 were exhibited. The color (red or blue) was proportional to the NES of the corresponding pathway in MRGPI-high or -low groups. **(C)** The expression of macrophage biomarkers in MRGPI-based groups. Mono/Macro subcluster was split into -high, -medium, and -low groups by the MRGPI. **(D)** Correlation between the MRGPI and different macrophages at the bulk-tumor level. *p < 0.05, **p < 0.01, ***p < 0.001. ns, non significant.

To characterize the TME of GBM, the CIBERSORT algorithm was employed to estimate the fraction of 22 immune infiltrations. 13 types of immune cells were retained after excluding cells with 0 value over half of the samples. As expected, the MRGPI-high group had significantly increased infiltration of monocyte, M2 macrophage, as well as Treg, NK cell (resting), and neutrophil (**Figure 4A**). Monocytes/macrophages in the TME are functionally pleiotropic and plastic (10). To further explore the association between MRGPI and the functional state of monocytes/macrophages, scRNA-seq dataset GSE131928 was employed in our study. As a result, 4 types of cells including malignant cell, Mono/Macro cell, CD8 T cell, and other unclassified cells were identified (**Supplementary Figure 8**; **Supplementary Table 3**). MRGPI was calculated based on the expression profile of the Mono/Macro (n = 3236) subcluster and GSEA analysis found the enrichment of inflammation, immunosuppression, and altered fatty acid and glucose metabolic signaling pathways in the MRGPI-high group of the Mono/Macro cells (FDR-q < 0.1)(**Figure 4B**, **Supplementary Table 2**), corroborating the results of the bulk-tumor level. The ribosome-related signaling pathways and cellular response to starvation were top enriched in the MRGPI-low group. In addition, cells in the Mono/Macro subcluster were further divided into high, medium, and low groups by the MRGPI. The expression of CD40, CD163, andMSR1 (CD204) was significantly increased in the MRGPI-high group (**Figure 4C**), indicating that MRGPI-high was associated with the alternative activation of macrophages. At the bulk-tumor level, multivariate regression analysis found a significant positive correlation between MRGPI and the fraction of M2 macrophages (**Figure 4D**), validating the association between MRGPI and alternative activation of TAMs in GBM TME.

### Expression of immune checkpoints associated with MRGPI

**Figure 5:**
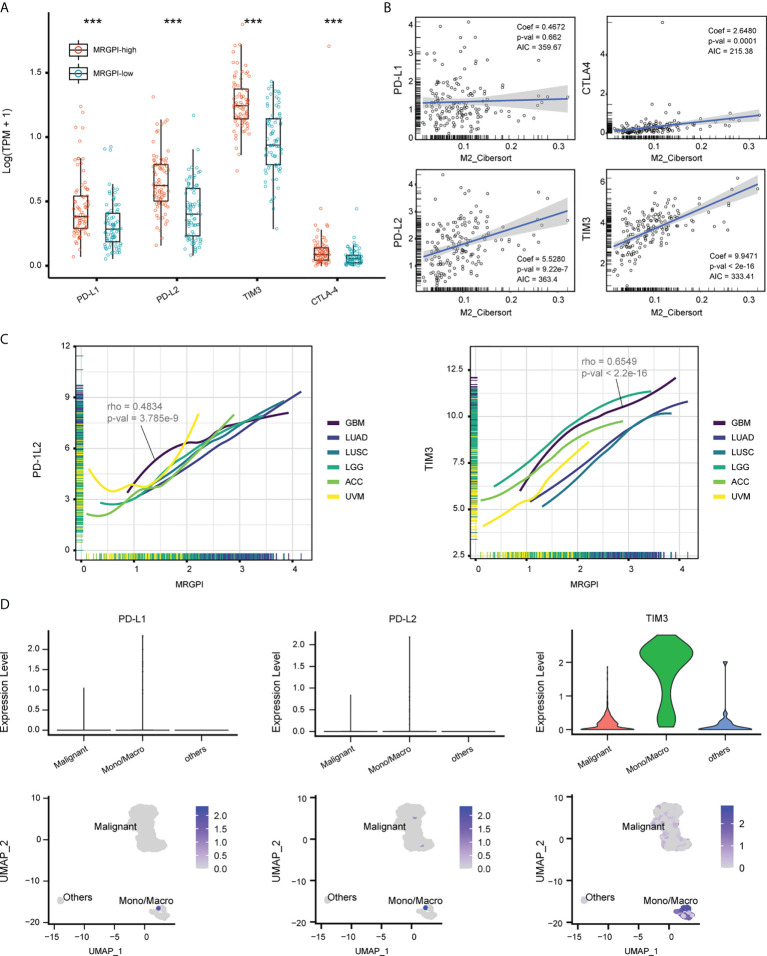
Expression of immune checkpoints associated with MRGPI. **(A)** The expression of PD-L1/2, TIM3, and CTLA-4 based on the TCGA cohort. **(B)** Multivariate regression analysis estimating the association between the expression of immune checkpoints and M2 macrophage faction. **(C)** Association between MRGPI and expression of PD-L2 and TIM3 on a pan-cancer scale. ACC and UVM that had sterile lymphocyte infiltration and LUAD and LUSC that had abundant lymphocyte infiltration were used as references. ACC, Adrenocortical carcinoma; UVM, Uveal Melanoma; LUAD, Lung adenocarcinoma; LUSC, Lung squamous cell carcinoma. **(D)** The expression of PD-L1/2 and TIM3 at a single-cell resolution based on GSE70630.

TAMs are known sources of immune checkpoints in TME, which contributes to the dysfunction of tumor-infiltrating CD8 T cells and frustration of anti-tumor immunity (12, 41). We first explored the expression pattern of immune checkpoints between groups. As a result, the expression of PD-L1, PD-L2, TIM3, and CTLA4 was significantly upregulated in the MRGPI-high group (**Figure 5A**), and the expression of these genes was positively correlated with the faction of M2 macrophages in the multivariate regression model, except for PD-L1 (**Figure 5B**). On a pan-cancer scale, the MRGPI was positively correlated with the PD-L2 and TIM3, whether in cancers that lack lymphocyte infiltration (ACC and UVM) or that are lymphocyte-rich (LUAD and LUSC) (**Figure 5C**). To further demonstrate, the scRNA-seq dataset GSE70630 was included and 3 types of cells were identified, including malignant cells, Mono/Macro, and other cells (mainly oligodendrocyte) (**Supplementary Figure 9**, **Supplementary Table 4**). At single-cell resolution, the expression of PD-L1, PD-L2, and TIM3 was predominantly located at the Mono/Macro subcluster (**Figure 5D**). Together, the MRGPI-based group identified a subset of GBM with increased expression of immune checkpoints in the TME, which may be associated with the M2 macrophages.

### Prediction of ICI responsiveness by MRGPI

**Figure 6:**
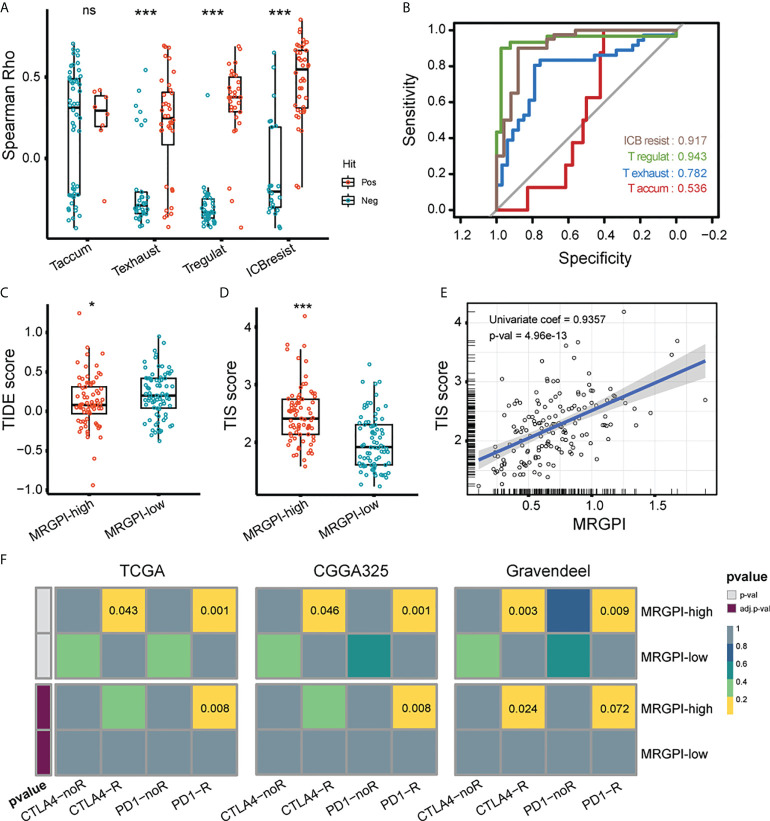
Potential of MRGPI in predicting ICB responsiveness. **(A)** Association between MRGPI and T cell dysfunction-related genes. To achieve this, 4 published gene signatures related to T cell dysfunction were collected and screened. The Spearman rho of positive (red) and negative (green) hit genes with MRGPI was exhibited. The difference in rho between positive and negative groups was compared through the two-sided Wilcoxon test. Taccum, T cell accumulation; Texhaust, T cell exhaustion; Tregulat, regulatory T cell; ICBresist, ICB resistance. **(B)** ROC curves evaluating the performance of MRGPI in predicting the positive and negative hit gene associated with T cell function. **(C)** TIDE score between the MRGPI-high and -low groups. **(D)** TIS score between the MRGPI-high and -low groups. **(E)** Correlation between the MRGPI and TIS score. **(F)** Submap algorithm manifested association between MRGPI-based groups and sample responsiveness to PD-1 and CTLA4 inhibitors. ***p < 0.001. ns, non significant.

Lastly, we evaluated the association between MRGPI and GBM responsiveness to ICI. There are published studies on tumor evasion and T-cell function, in which key genes have been screened and collated by Peng et al. (**Supplementary Table 5**) (37, 42–45). Briefly, the positive or negative hits are defined as genes upregulated or downregulated in the shRNA screen. We analyzed the association of the positive and negative hit genes with MRGPI using the spearman correlation test and rho instead of Peng et al. using the Cox-ph model test and d values. As a result, MRGPI had a significantly increased correlation with genes that are functionally related to T cell exhaustion, T regulatory cell, and ICB resistance (anti-CTLA4) (**Figure 6A**). The ROC curves indicated that the MRGPI gave the best performance in predicting T regulatory and ICB resistance (anti-CTLA4 treatment) genes (**Figure 6B**), indicating the association between MRGPI and T cell dysfunction. TIDE dysfunction score is associated with T cell dysfunction for lymphocyte-rich tumors and the prevention of T cell infiltration for lymphocyte-poor tumors at transcriptome level (37). As a result, the MRGPI-high group had a significantly decreased TIDE score (**Figure 6C**). Given that GBM is a kind of tumor lacking lymphocytic infiltration, a reduced TIDE score may indicate that the MRGPI-high group is less efficient at suppressing local antitumor immune responses by impeding T cell infiltration. Meanwhile, the TIS score was an mRNA metric associated with interferon gamma-mediated PD-1 signaling that was also associated with sample response to ICI (46). We found that the MRGPI-high group had significantly increased TIS scores, and a robust correlation was found between the MRGPI and TIS score (univariate regression coef = 0.936) (**Figure 6D, E**). Then, we employed the submap algorithm to classify sample responsiveness to PD-1 and CTLA4 inhibitors, with reference to a cohort of cutaneous melanomas treated with ICI inhibitors (47). With the 3 GBM cohorts corroborating each other, the MRGPI-high group showed potential responsiveness to anti-PD1 treatment (FDR-q < 0.01) (**Figure 6F**). Therefore, these results suggested that blocking the PD1/PD-L1 axis may be applicable to the MRGPI-high group.

## Discussion

A great deal of research is currently dedicated to improving the treatment of GBM, and the efficacy of ICIs, although remains limited, is still a promising treatment modality. Given that the overall response rate of GBM to ICI remains dismal, it is crucial to determine who will benefit from the therapy. Several studies are devoted to develop biomarkers related to the prognosis and treatment effecacy of GBM from a tumor ontology or TME perspective, but the results are not yet satisfactory (48). Recent transcriptome-based achievements in immunophenotyping of gliomas and transcriptomic characterization of macrophage polarization provide the basis for screening genetic metrics of clinical implication from TAMs.

TAMs are the main immune cells in the GBM microenvironment, accounting for up to 30% or more of the tumor tissue, and are decisive for several endowments of the TME (49). The role of TAMs in remodeling the extracellular matrix through MMPs and in inducing angiogenesis through the production of VEGF/EGF is well-documented (10, 50). With the rise of immunotherapy, the immunological features of the TME have received increasing attention and thus driven the understanding of how TAMs abet the immunosuppressive TME (11, 51). Previous studies have reported the role of the PI3K-Akt-mTOR signaling pathway in promoting tumor cell proliferation and rewiring tumor metabolism (40). In terms of microglia, mTOR-mediated activation of STAT3 and NK-κB is associated with an immunosuppressive phenotype. Inhibition of mTOR then promoted an inflammatory microenvironment and the proliferation of CD4^+^ and CD8^+^ T cells (11). In addition, the PI3K/Akt signaling pathway has been associated with dysregulation of lipid metabolism. The rewired lipid metabolism is associated with the hydrolysis of arachidonic acid from the membrane, and the production of bioactive eicosanoid derivatives such as PGE_2_ and LTB_4_ (40, 52). TAMs are responsible for the production of such inflammatory response mediators in the TME (39). Notably, TAMs are involved in the dysfunction of CD8 T cells by expressing multiple immune checkpoints and the induction of other cells in the TME in expressing immune checkpoints (13, 15). On this basis, we consider that the construction of gene signatures associated with TAMs may also be useful for immunotherapy. There are two concerns, however. The measurement of immune checkpoints from mRNA expression levels alone may be inadequate, and future studies should take into account the tissue and cellular localization of immune checkpoints. As well, the M1 and M2 typing of TAMs is oversimplistic (53), and identifying subtypes of TAMs that specifically express immune checkpoints would promote precision oncology.

MRGPI is composed of 5 genes, including GPR84, NCF2, HK3, LILRB2, and CCL18. G protein-coupled receptor 84 (GPR84) is a receptor for medium-chain free fatty acids with carbon chain lengths of C9 to C14 (54). It is mainly expressed by monocytes, macrophages, and neutrophils and promotes inflammatory responses by activating ERK and elevating levels of intracellular Ca^2+^ and IP (55). Inflammatory colon tissue from patients who suffered from ulcerative colitis is filled with large numbers of GPR84-positive macrophages, whose involvement in the inflammatory response may depend on the activation of NLRP3 inflammasome (56). Neutrophil cytoplasmic factor 2 (NCF2) encodes a subunit of the multi-protein NADPH oxidase complex, which is involved in the bursting of superoxide in neutrophils. Single nucleotide polymorphisms in NCF2 are associated with diminished NADPH oxidase activity, which in turn is involved in the pathogenesis of systemic lupus erythematosus (57). In addition, Wang et al. identified that NCF2 was associated with poor prognosis in GBM by WGCNA analysis (58). Xu et al. reported that the inhibition of glioma cell growth by miR-524 was achieved by targeting NCF2 (59). Hexokinase 3 (HK3) phosphorylates glucose to produce glucose-6-phosphate, which is then imported into the glucose metabolic pathway. In non-small cell lung cancer, HK3 expression correlates with immune cell infiltration and tumor sensitivity to Pembrolizumab (60). In addition, HK3 overexpression also promotes prostate cancer, acute myeloid lymphoblastic leukemia, and colon tumors (61–63). Leukocyte immunoglobulin-like receptor B2 (LILRB2) is a member of the leukocyte immunoglobulin-like receptor (LIR) family and encodes a protein that belongs to the B subfamily of LIR receptors. One ligand for LILRB2 is HLA-G. The level of serum soluble HLA-G is negatively associated with survival of glioma patients (64). Previous study haa highlighted the function of HLA-G in promoting immune escape from tumors, but the exact mechanism has not been elucidated (65). Our results suggested that HLA-G may regulate the function of TAMs in GBM *via* binding to LILRB2. C-C motif chemokine ligand 18 (CCL18) encodes the cytokine CCL18 which has bactericidal and T lymphocyte chemotactic effects. CCL18 promotes the invasion and epithelial-mesenchymal transition of a variety of tumor cells, including squamous skin cancer, breast cancer and liver cancer, through interaction with its receptor PITPNM3 (66, 67). A recent study indicate that CCL18 derieved from TAMs are vital in promoting glioma progression. GBM releases extracellular vesicles containing CCL18 to allow surrounding tumor cells to acquire resistance to temozolomide (68). Notably, in addition to being expressed at the mRNA level and having functional implications, proteins encoded by these genes showed a tendency to be differentially expressed between tumor core and margin. Through immunohistochemical staining, we found that the proteins encoded by GPR84 and NCF2 were significantly increased in tumor core, further suggesting a pro-tumoral role of these genes. However, more samples were needed in future study. Taken together, MRGPI is a prognostic marker associated with the inflammatory response mediated by TAMs.

TME is a sophisticated multi-cellular collaborative system. In the central nervous system, the GBM microenvironment is unique due to the presence of the brain-blood barrier, the type and number of resident immune cells, the type of extracellular matrix, and the specific immunological properties of brain tissue (49, 69). When exploring the association between the MRGPI-based group and immune infiltration in the GBM microenvironment, we found that the MRGPI-high group contained more immune cells involved in the inflammatory response, including monocytes, M2 type macrophages, and neutrophils, while there was little difference in the content of lymphocytes associated with the adaptive immune response. The type of inflammatory response affects the growth of tumor cells, and it is generally accepted that acute inflammatory responses have an overall inhibitory effect on tumor cells, but chronic inflammatory responses promote malignancies (70, 71). However, the toxic effects of inflammation on neurons would be very similar, i.e. inducing demyelination and death of these cells (49). In addition, as the volume of the cranial cavity is limited, edema and increased intracranial pressure caused by a strong or persistent inflammatory response can be fatal (72, 73). These features of the central nervous system may hinder the application of immunotherapies: the immune response cannot be enhanced indefinitely but should be kept within a tolerable range. Determining such a range should take into account individual factors, including gender, age, the fullness of brain tissue on imaging, and neurological function. This may suggest that, in addition to the development of biomarkers to predict the sensitivity of GBM patients to immunotherapy, markers to predict the upper limit of patients’ tolerance to immune responses are also needed.

In characterizing the molecular mechanisms associated with the MRGPI-based group, we found increased eicosanoid metabolic response activity in the MRGPI-high group, particularly in the leukotriene metabolic pathway. Leukotrienes are derived from the processing of arachidonic acid by lipoxygenases and the end products include LTA_4_, LXA_4_, LTB_4,_ and LTC_4_ (74). The origin of leukotrienes in inflammation is complex and can be produced by a single cell with an entire enzyme system or by multiple cells working in collaboration (74, 75). Similar to prostaglandins, leukotrienes are undoubtedly potent inflammatory factors involved in the development and progression of asthma, rheumatoid arthritis, and age-related central nervous system disorders (76). However, their role in tumors remains controversial. LTB_4_-mediated chronic inflammation contributed to the growth of transplanted melanoma and blocking LTB_4_ partially inhibited such effect. Interestingly, it was not the well-known pro-tumoral COX-1/2 that promoted tumor cell proliferation in this model (77). Jung Yeon Lim et al. reported that *in vitro* interference with 5-LO or FLAP expression using MK886 or siRNA induced apoptosis of glioma cells, suggesting a role for leukotrienes in promoting glioma proliferation (78). In TME, a range of 5-LO expressing stromal and immune cells have also been found to promote tumor metastasis, recruit other inflammatory cells, and create a chronic inflammatory response to accelerate tumor progression (79). Therefore, prohibiting leukotrienes, especially LTB_4_, may be promising for both targeting GBM cells and TME.

The TIDE score is an integrated metric evaluating T cell dysfunction in tumors with high cytotoxic T lymphocyte infiltration and the inhibitory of T cell infiltration in tumors with low cytotoxic T lymphocyte infiltration and is a valid marker in predicting ICI benefits (37). Samples with increased TIDE scores have a higher potential for immune evasion, indicating that they are less likely to benefit from ICI (37). However, the original study found that GBM lacks genes that interact with the CD8 T cell mRNA metric in the Cox-ph model, which may reduce the effectiveness of TIDE for GBM. Besides, the TIS score consists of 18 genes associated with interferon-gamma. As interferon-gamma upregulates PD-L1 *via* the JAK-STAT pathway, the TIS score is also a highly effective prognostic and predictive marker for ICI benefit in pan-cancer analysis (46, 80). In our study, no significant differences in the level of lymphocyte infiltration emerged between MRGPI groups, but significant differences in the expression of immune checkpoints were found, suggesting that MRGPI may distinguish between GBM samples that avoid immune attack *via* the immune checkpoint pathway. Such a presumption would also fit with the TIS score assuming the presence of an activated but suppressed adaptive immune response in the tumor sample. To further validate these results, we employed a subclass mapping method algorithm to categorize GBM samples according to the expression profile of cutaneous melanoma and the corresponding responsiveness to ICI treatment, and the results again supported the sensitivity of the MRGPI-high group to PD1 blockade treatment. Therefore, although GBM is a ‘cold tumor’ with low immune cell infiltration and the CNS is compatible with multiple immunosuppressive mechanisms, MRGPI is still a potential marker that identifies a class of GBM characterized by immune checkpoint-mediated immunosuppression.

In conclusion, MRGPI is a promising prognostic biomarker. The MRGPI-based group may help to differentiate immunological features and serve as a potential response indicator for immunotherapy.

## Data availability statement

Datasets included in this study can be found on the website described in the material and methods, and they can be acquired from the corresponding authors upon reasonable request.

## Ethics statement

The studies involving human participants were reviewed and approved by The Ethics Committee of the Ethics Committee of Zhejiang Provincial People’s Hospital. The patients/participants provided their written informed consent to participate in this study.

## Author contributions

HJ and ZL conceived and designed the study. HJ collected and analysed the data and generated the figures. HJ drafted the manuscript. ZL and HS revised the manuscript. NW provided analytical support. FW and YL conducted the immunohistochemical staining. CY, SH, and YL supervised the study. All authors contributed to the article and approved the submitted version.

## Funding

This work is funded by Zhejiang Provincial People’s Hospital Talent Introduction Project (No. C-2021-QDJJ03-01)

## Acknowledgments

We thank Miss Zhou Jie and Mr. Zhang Jing for providing additional statistical support.

## Conflict of interest

The authors declare that the research was conducted in the absence of any commercial or financial relationships that could be construed as a potential conflict of interest.

## Publisher’s note

All claims expressed in this article are solely those of the authors and do not necessarily represent those of their affiliated organizations, or those of the publisher, the editors and the reviewers. Any product that may be evaluated in this article, or claim that may be made by its manufacturer, is not guaranteed or endorsed by the publisher.
