# Supplementary material for: Novel macrophage-related gene prognostic index for glioblastoma associated with M2 macrophages and T cell dysfunction
Source: Front Immunol. 2022 Sep 13;13:941556. doi: 10.3389/fimmu.2022.941556 (PMC9513135; doi:10.3389/fimmu.2022.941556)
Supplement: Supplementary file 6 [file DataSheet_1.docx]

Supplementary Material

# Supplementary Figures


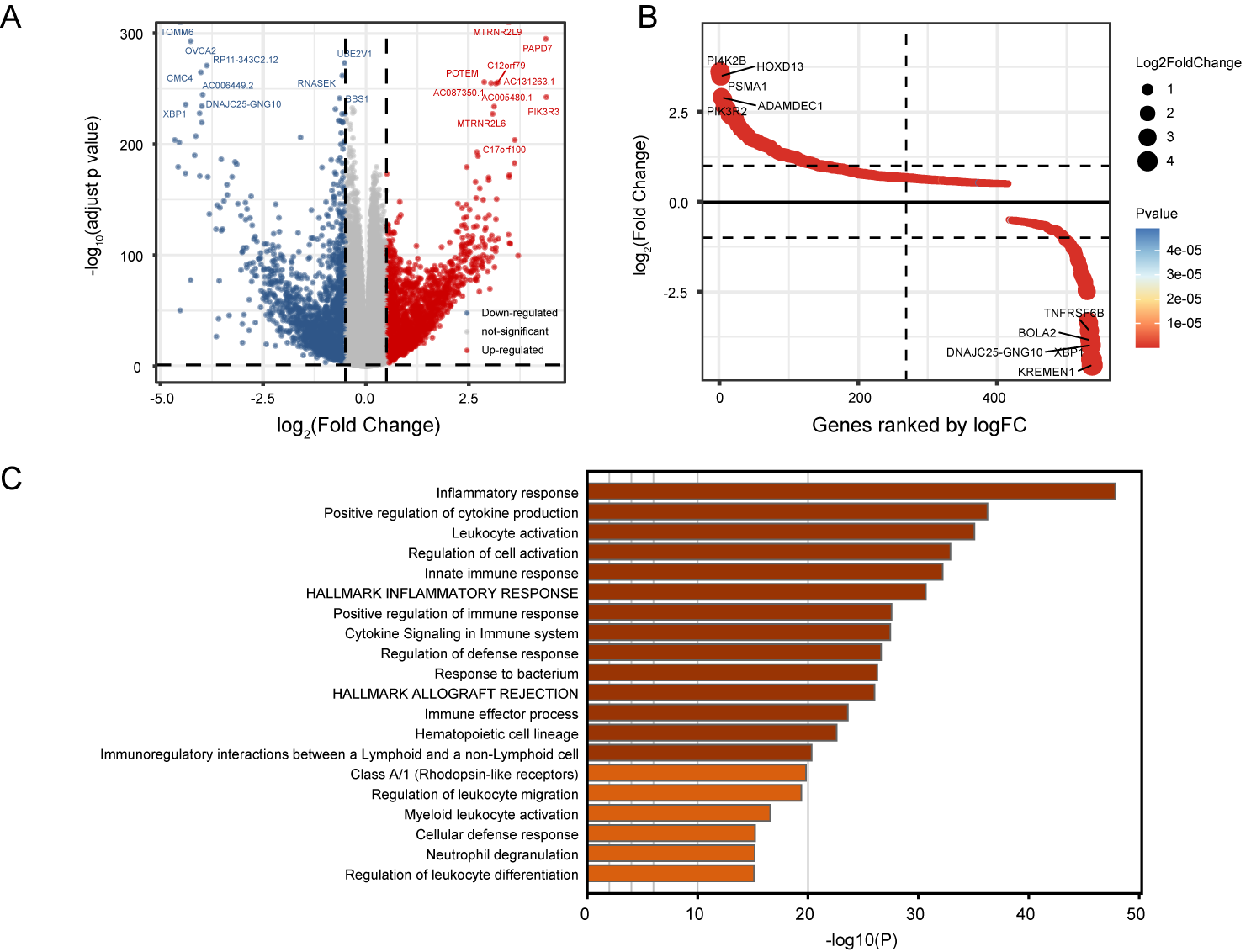


**Supplementary Figure 1. Identification of differentially expressed macrophage-related genes.** (**A**) 3528 DEGs between GBM and normal samples. |logFC| >= 0.5 and adj p-val < 0.05 were set as the cut-off. The top 10 upregulated and downregulated genes were indicated. (**B**) 537 DEGs related to macrophage. The top 5 upregulated and downregulated macrophage bona fide genes were indicated. (**C**) Functional enrichment analysis of the 537 macrophage-related DEGs. GO biological process, KEGG, HALLMARK, PID, WP, Biocarta, and HP pathways were involved.


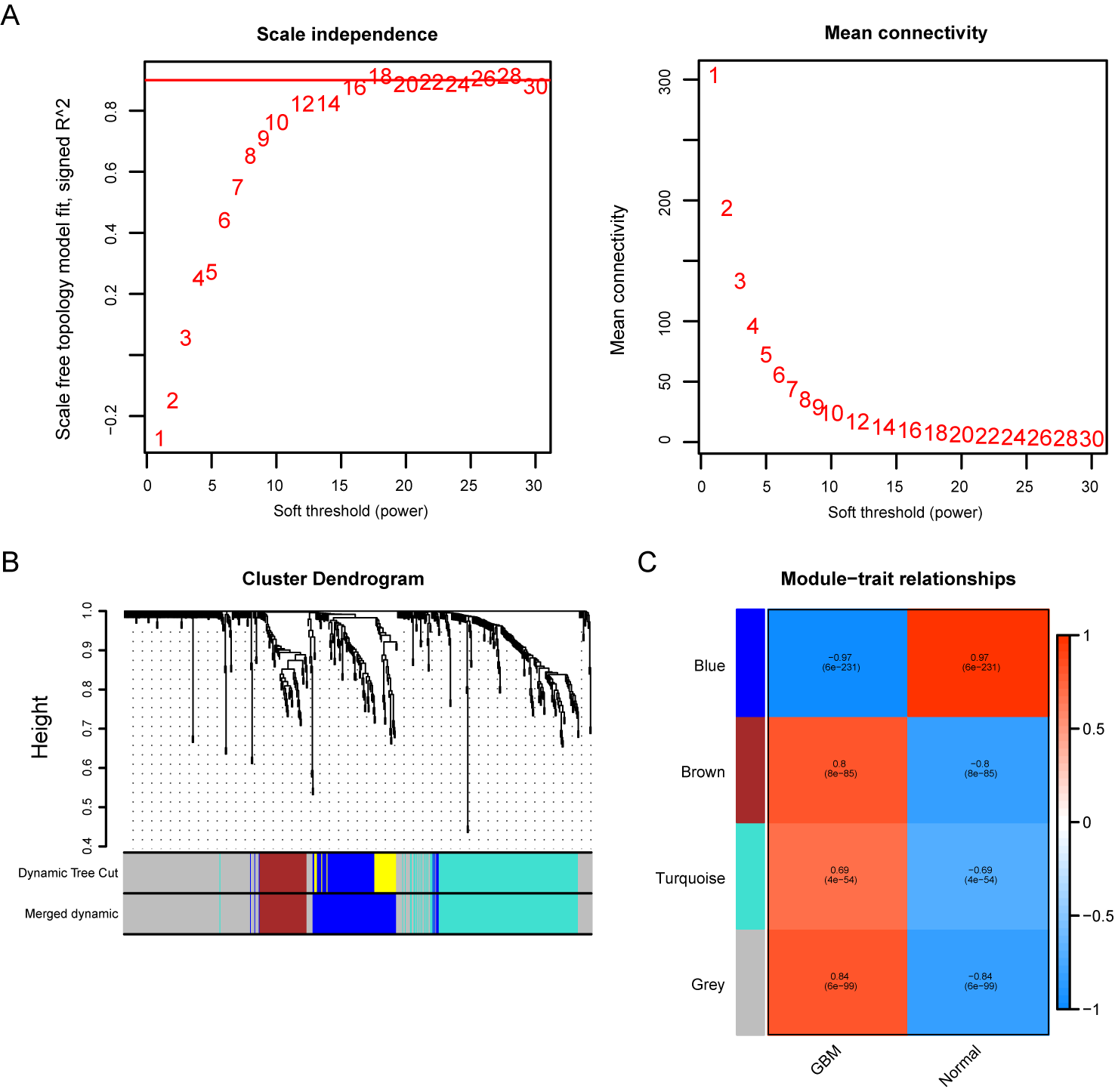


**Supplementary Figure 2. Identification of macrophage-related hub genes.** (**A**) Selection of optimal soft threshold for modularization of the 537 differentially expressed macrophage-related genes. (**B**) Hierarchical clustering of these genes with a soft threshold β = 16. (**C**) Gene modules and their Pearson correlation with clinical features.


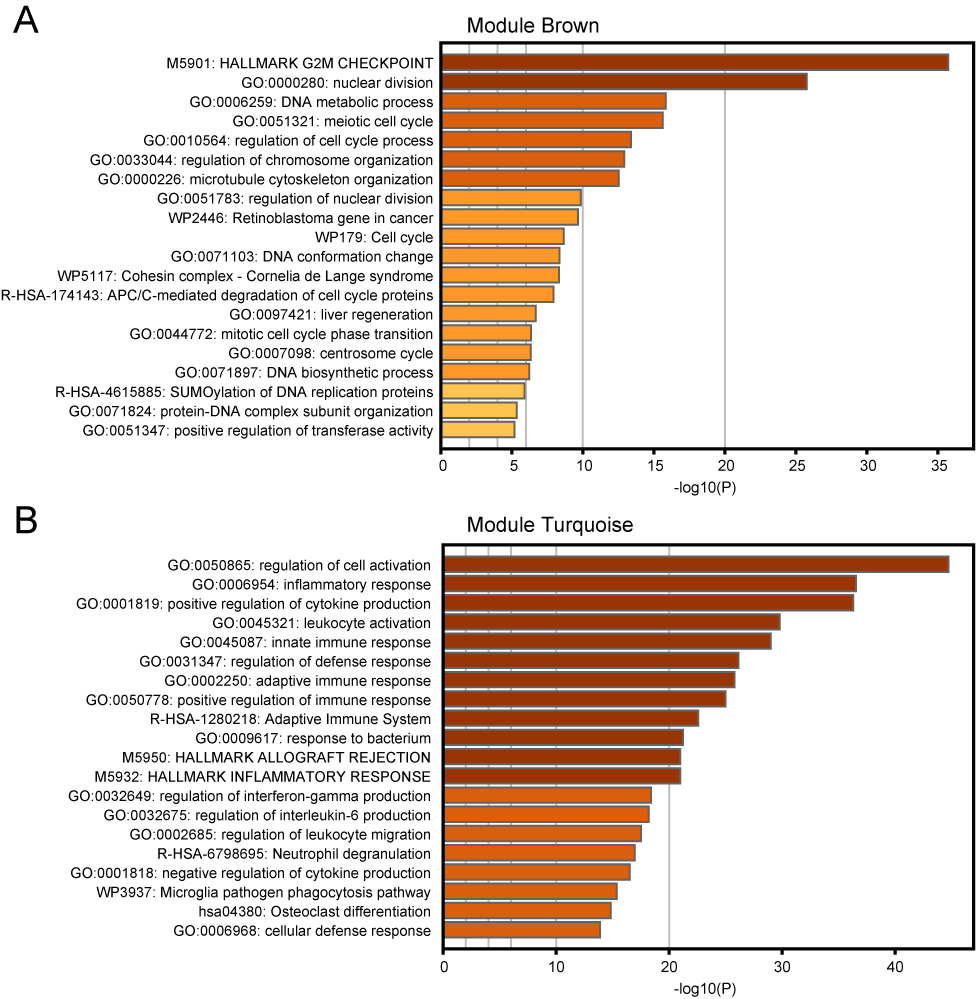


**Supplementary Figure 3.** Functional enrichment analysis of 232 genes involved in (**A**) module brown and (**B**) module turquoise.


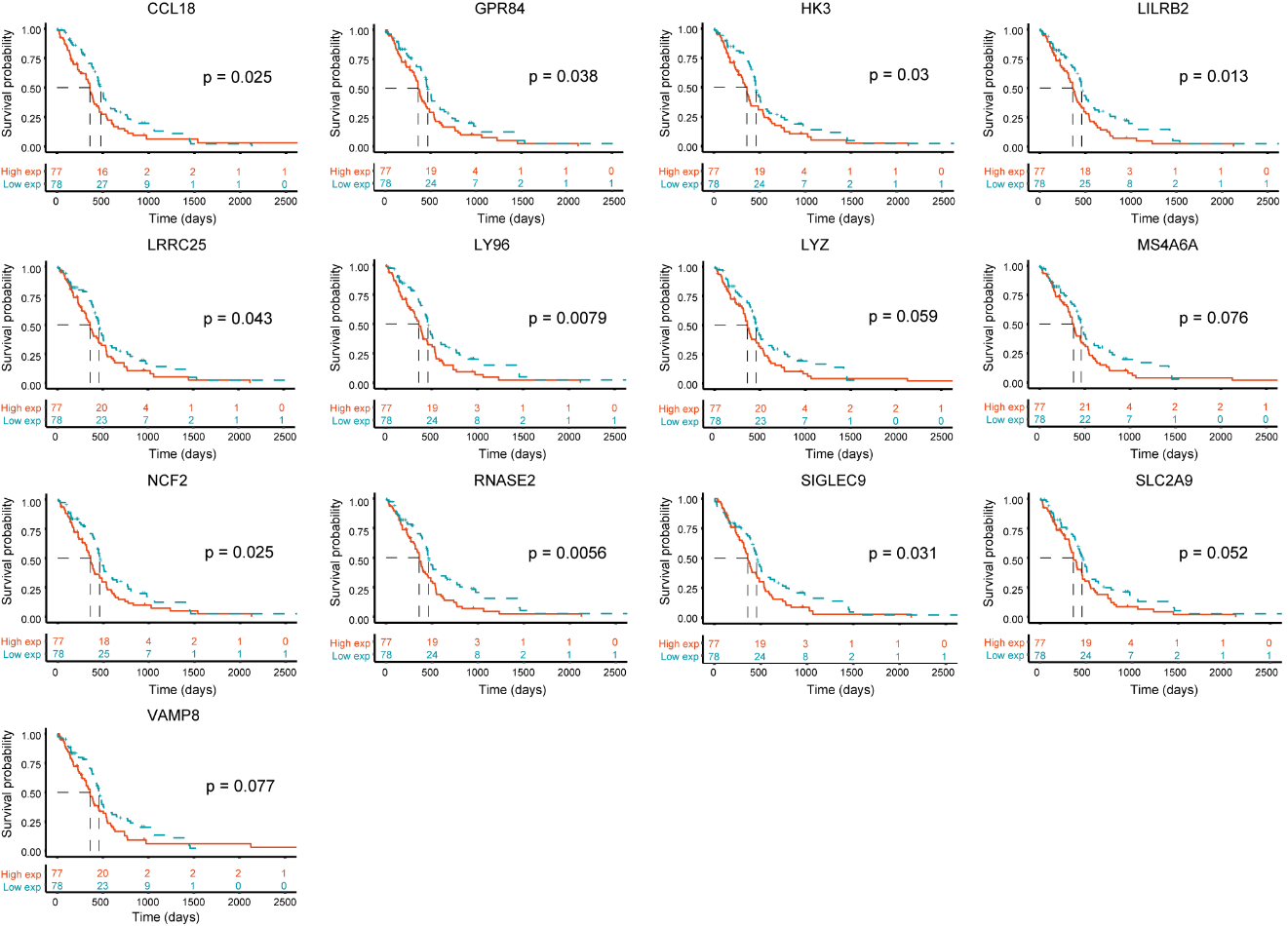


**Supplementary Figure 4.** K-M analysis for screening of the hub genes in module brown and turquoise. 13 of the 97 genes of prognostic significance were exhibited. K-M: Kaplan-Meier.


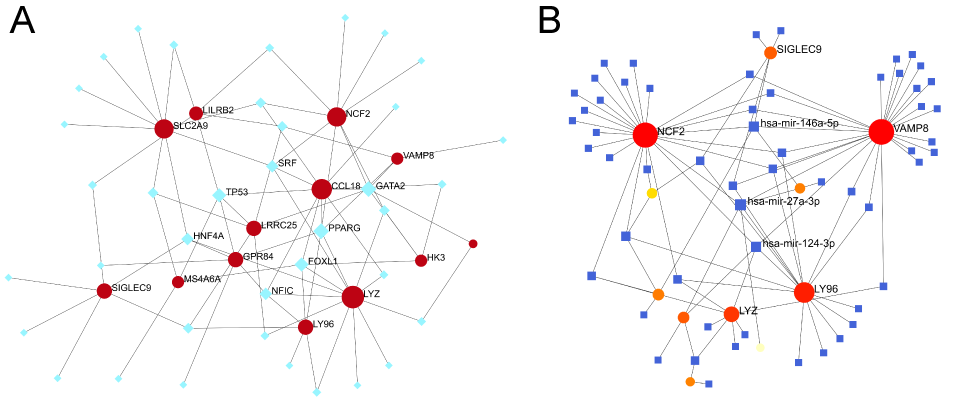


**Supplementary Figure 5.** Network analysis summarized the sophisticated interactions of the 13 genes of independent prognostic significance with (**A**) transcriptional factors and (**B**) miRNAs. The size of the node was positively correlated with its connectivity degree.


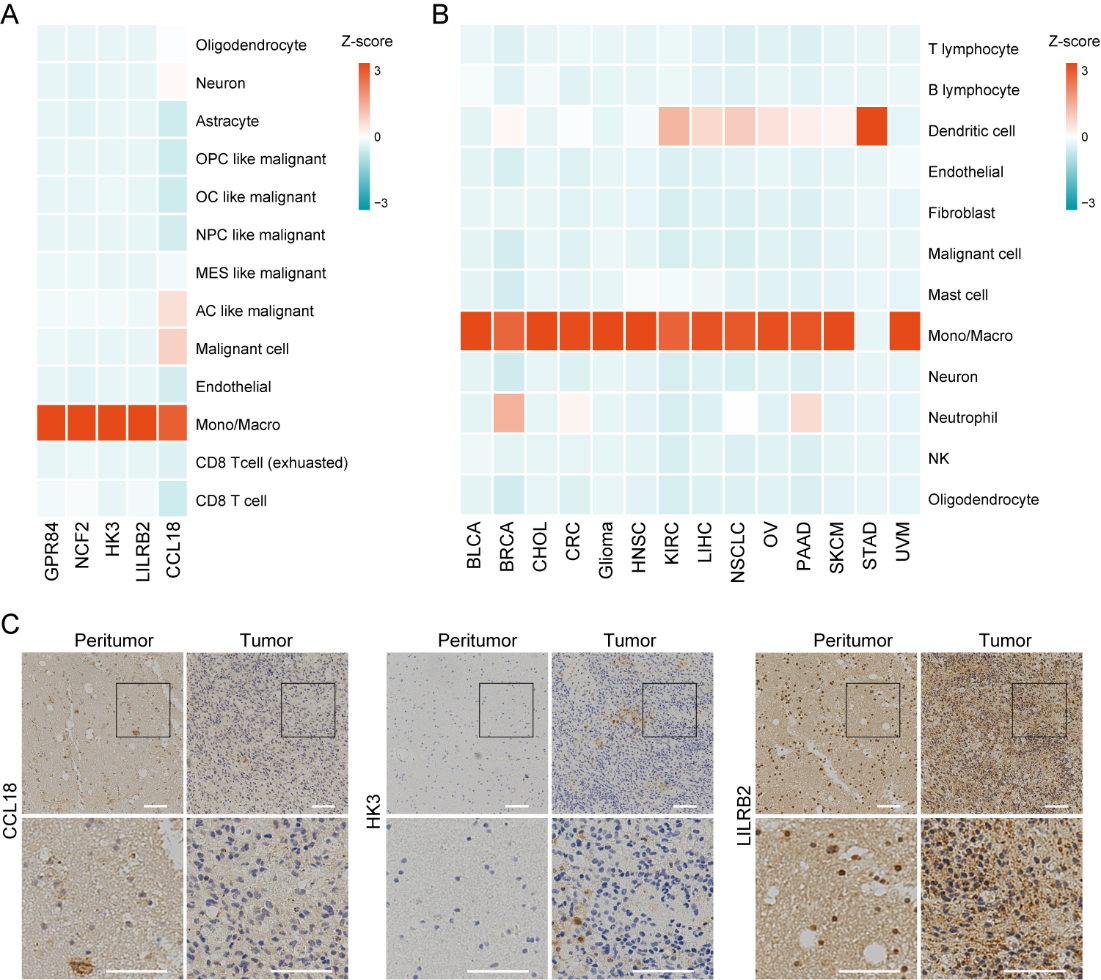


**Supplementary Figure 6.** **Cellular location of the 5 selected macrophage-related hub genes.** (**A**) The average expression (log transformed TPM) of each gene in several subtypes of cells in 12 single-cell glioma expression profiles. (**B**) The cellular location of the average expression of the 5 gene in a pan-cancer scale. The cell subtypes were integrated as follows. Malignant cell = Malignant + AC-like + ME-like + OC-like + OPC-like + NPC-like; T lymphocyte = CD8 T + CD8 Tex + Tprolif + Treg; B lymphocyte = B + Plasma; Fibroblast = Fibroblast + Myofibroblast. (C) The immunohistochemical staining of CCL18, HK3 and LILRB2-encoded proteins in tumor cores and margins (Scale bar, 100μm).


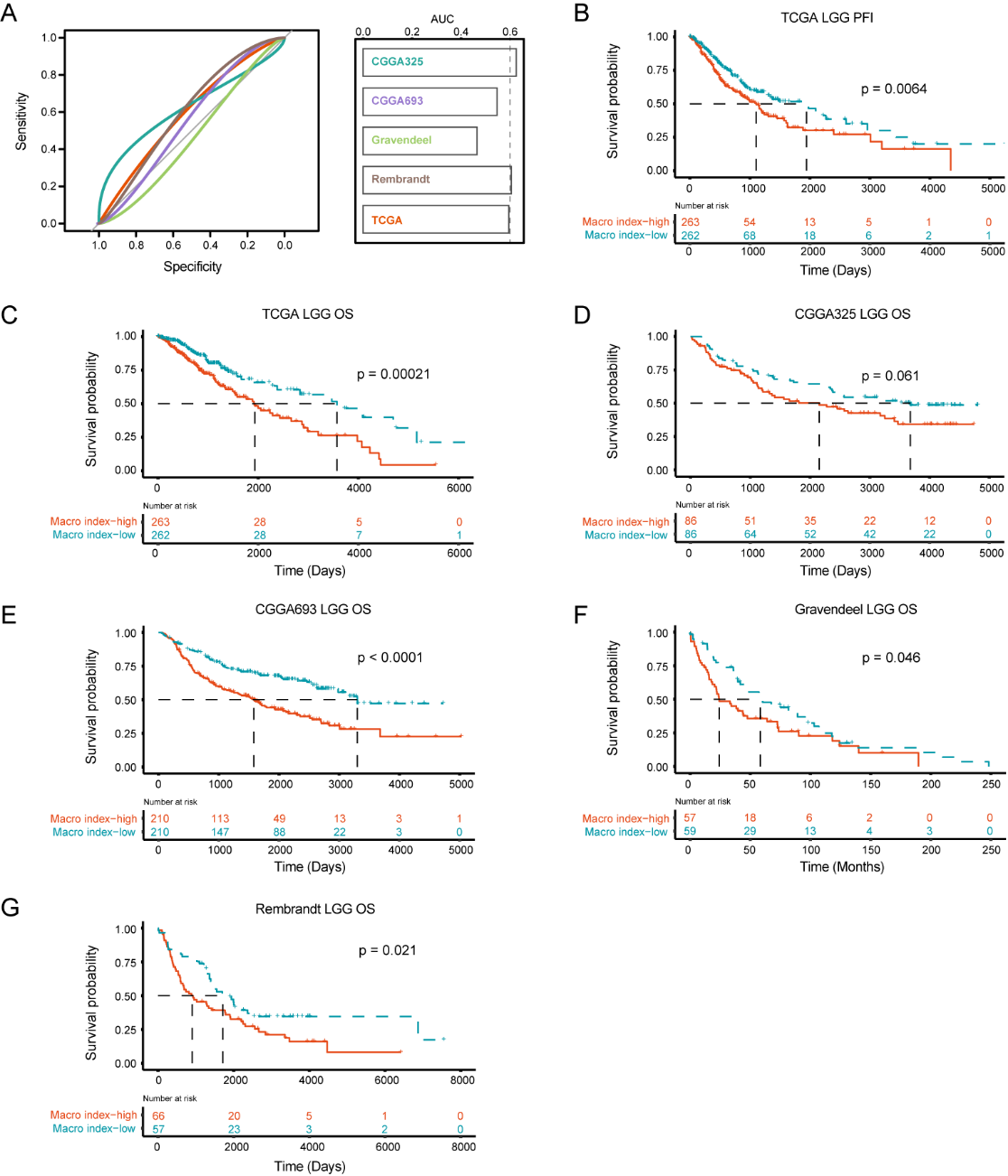


**Supplementary Figure 7.** **The prognostic significance of MRGPI in LGG and GBM.** (**A**) The prognostic predictive performance of the MRGPI based on both training and validation data sets. (**B-G**) K-M analysis suggested that the MRGPI was also a robust predictive biomarker for LGG, with the MRGPI-high stably indicating an unfavorable outcome.


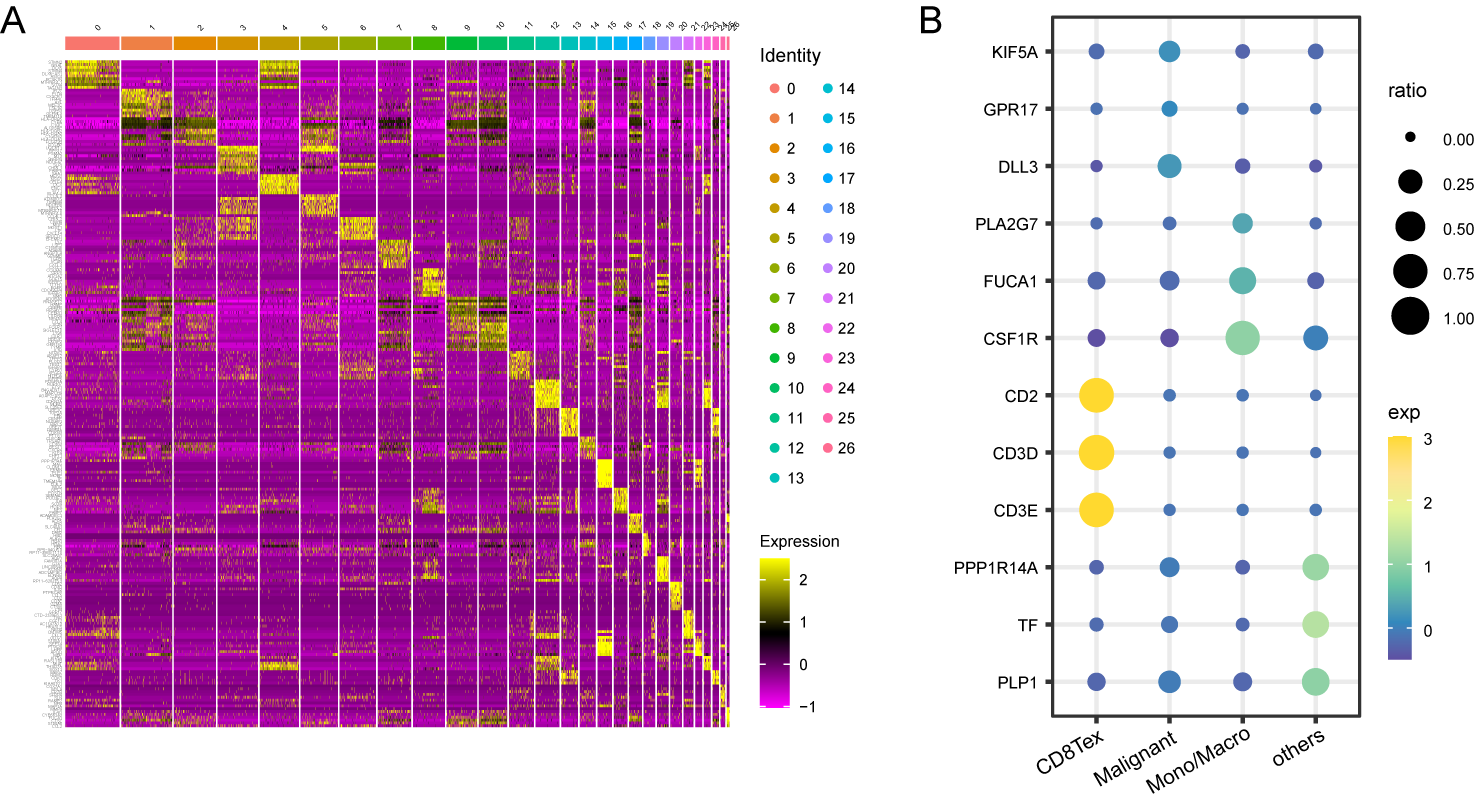


**Supplementary Figure 8.** **Identification of subclusters and cell types of the GSE131928.** (**A**) Clusters identified by Seurat at a resolution of 0.5. (**B**) Cell types annotated based on the expression of marker genes.


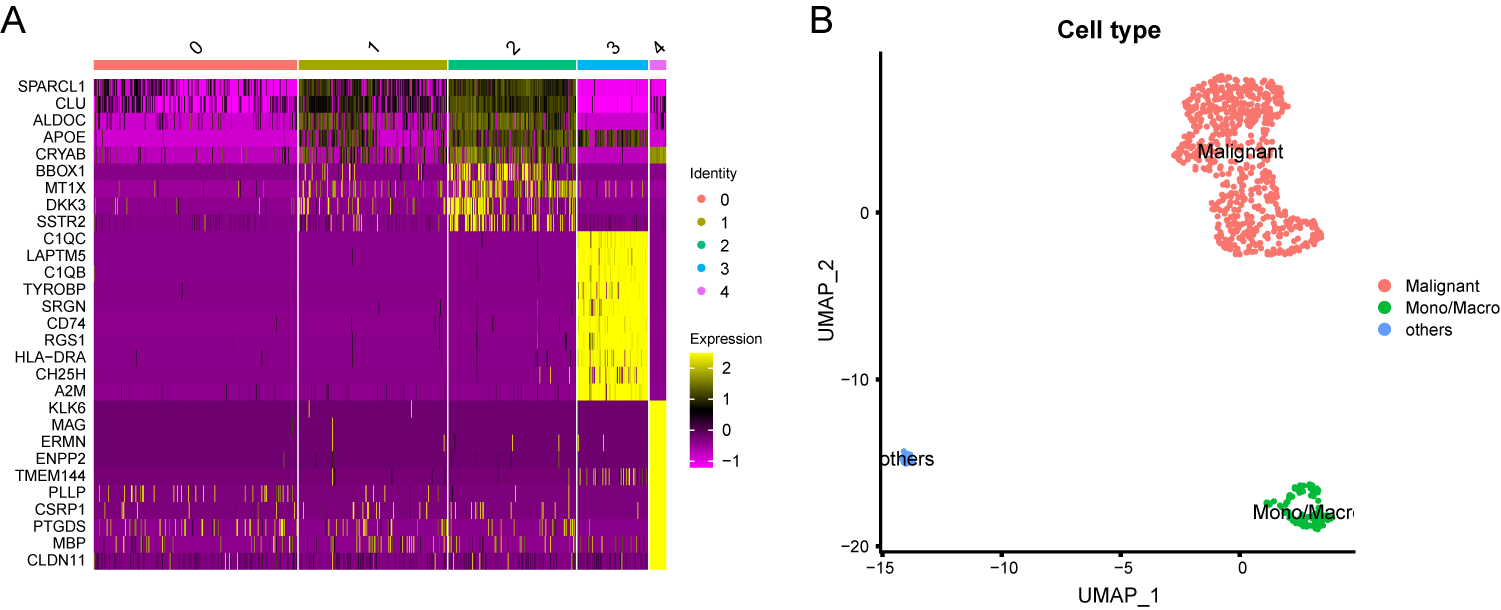


**Supplementary Figure 9. Identification of subclusters and cell types of the GSE70630.** (**A**) Clusters at the resolution of 0.5. (**B**) Distribution of cells identified.
